# Accurate Diagnosis of Severe Hypospadias Using 2D and 3D Ultrasounds

**DOI:** 10.1155/2016/2450341

**Published:** 2016-09-27

**Authors:** Vanesa Rodríguez Fernández, Carlos López Ramón y Cajal, Elena Marín Ortiz, Nerea Sarmiento Carrera

**Affiliations:** ^1^Service of Obstetrics and Gynecology, Álvaro Cunqueiro Hospital, Vigo, Spain; ^2^Service of Pediatrics, Álvaro Cunqueiro Hospital, Vigo, Spain

## Abstract

The hypospadias is the most common urogenital anomaly of male neonates but the prenatal diagnosis of this is often missed before birth. We present the prenatal diagnosis of a severe penoscrotal hypospadias using 2D and 3D ultrasounds. 3D sonography allowed us the best evaluation of the genitals and their anatomical relations. This ample detailed study allowed us to show the findings to the parents and the pediatric surgeon and to configure the best information about the prognosis and surgical treatment.

## 1. Introduction

Hypospadias is a congenital anomaly in the development of the urethra, in which the urethral meatus is abnormally positioned with a ventral opening in the surface of the penis [1].

Despite hypospadias being the most common congenital defect of the male external genitalia, it is often missed on prenatal ultrasound. Most diagnosed cases are severe forms, those associated with other anomalies or those with a family history of hypospadias.

We present a case of a severe penoscrotal hypospadias diagnosed prenatally using 2D and 3D ultrasound, which was crucial for an accurate diagnosis and proper parental advice.

## 2. Case Report

A 26-year-old woman, primigravida, with no significant past medical history was referred to our Prenatal Diagnosis Unit because in the 21-week ultrasound the sex was not clearly differentiated. Due to the ambiguity of the genitalia, a fetal karyotype in maternal blood was requested and it revealed a normal male karyotype (46XY). We performed a detailed fetal examination using ultrasound equipment Epiq 7 (Phillips Medical Systems, Bothell, WA, USA) with abdominal probes C5-1 and xMATRIX 6-1.

The fetal biometric measurements correlated with his estimated gestational age and showed no fetal anatomic abnormality except in the external genitalia.

**Figure 1:**
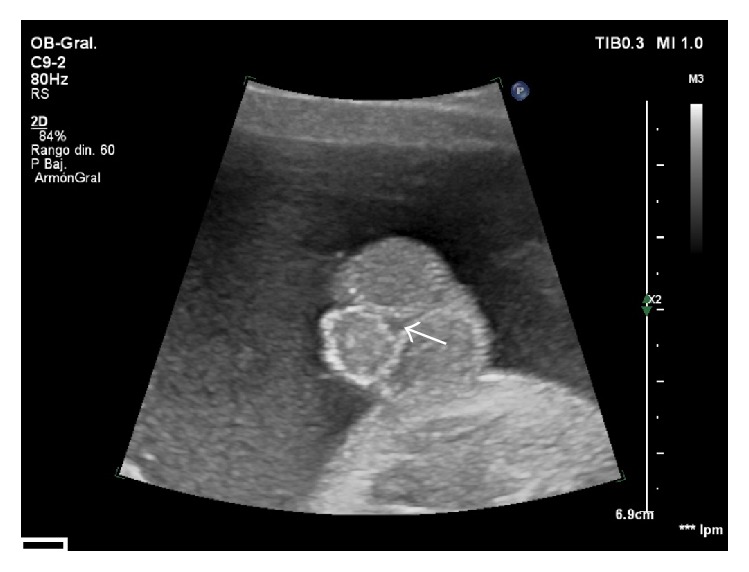
Two-dimensional image where we can see a small penis ventrally oriented and located between the scrotal folds, which include both testes. In this projection it is possible to see the anomalous localization of the meatus (arrow).

**Figure 2:**
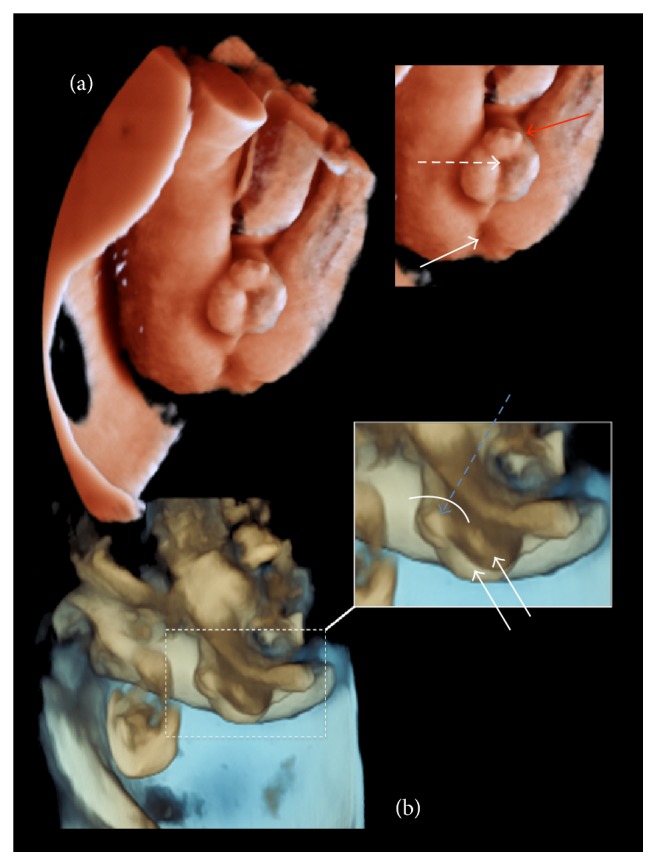
Three-dimensional representation of the genital surface. The configuration of the penis and its anatomical relations is shown in ample detail. (a) It is possible to detect the urethral meatus in the base of the penis (dashed arrow) and the surplus prepucial tissue at the tip forming the cap (red continuous arrow). We can also notice its relation with the perineum (the continuous arrow points to the anus). (b) In the transversal view it is possible to see the shortened penis (blue dashed arrow) with the chordee (curved line) and the surplus foreskin. We can also observe the two scrotal folds with both testicles inside (double arrow).

**Figure 3:**
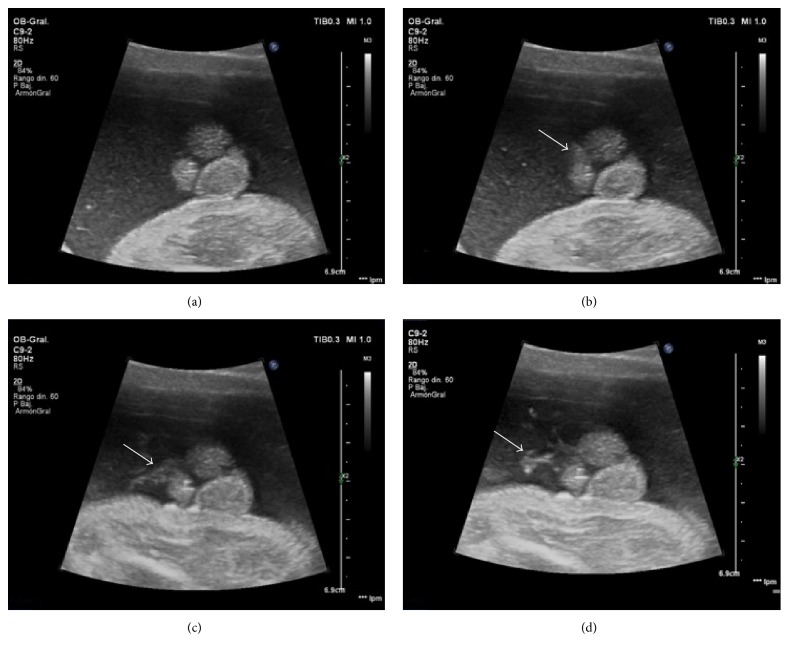
Consecutive sonographic images of the fetal micturition, achieved after external stimulation, which allow us to see the ventral urinary jet (arrows) near the penile base.

In the external genitalia a shortened penis is observed with a ventral angulation between the two scrotal folds. The two testicles were properly descended into the scrotum (Figure 1). 3D ultrasound was used to obtain a more precise diagnosis (Figure 2). In the volumetric study and surface rendering mode we clearly visualized a shortened and curved but correctly formed penis, with a surplus foreskin at the tip forming the prepucial cap. We can guess the location of the urethral meatus, in the most anterior part of interscrotal fold. The diagnosis is supported by viewing the fetal micturition, showing a ventral jet from the shaft of the penis, near the base (Figure 3). The exploration was compatible with a third-degree hypospadias.

The remaining ultrasounds performed during pregnancy showed no other relevant findings except for a fetal weight at the lower limit.

**Figure 4:**
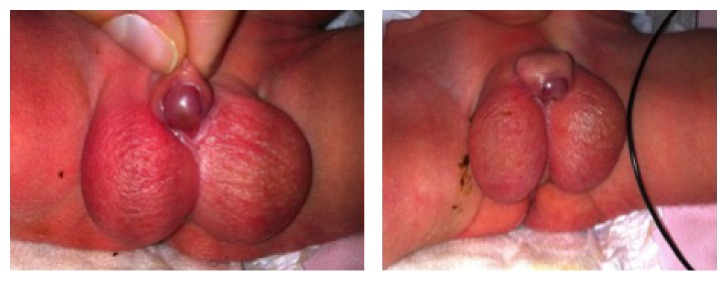
Images of the external genitalia of the newborn, which confirm the third-degree hypospadias. We can see the urethral meatus located in penoscrotal union, a bifid scrotum, and a curved penis with a foreskin forming a dorsal cap and a cleft ventral glans.

The patient was admitted at 37th week of gestation for a hypertension study. At 37 + 1 weeks a male of 2100 grams (percentile <3) was delivered by an induced vaginal delivery. The newborn was admitted to the Neonatal Unit because of low weight for gestational age. The exploration of the external genitalia confirmed the diagnosis of severe hypospadias with the urethral meatus at the base of the penis, almost interscrotal, a severe curvature, and a bifid scrotum with the testes in the bag, without other associated anomalies (Figure 4).

## 3. Discussion

We present a case of hypospadias where the surface rendering and multiplanar mode allowed us to ascertain its severity and the perineal anatomical relationships, in order to advise parents on the expected surgical outcomes.

Hypospadias is one of the most common congenital anomalies, with an estimated prevalence between 0.2 and 4.1 per 1000 live births of both sexes [2]. It is defined by a failed development of the spongy urethra and ventral foreskin [3]. The incomplete fusion of the urethral folds results in anomalous location of the meatus at some point of the ventral side of the penis, instead of the tip. The foreskin does not fuse on the ventral side, because of nonunion of ectodermal folds, being redundant dorsally. It is often associated with a ventral curvature of the penis (also known as chordee), caused by atresia of the corpus spongiosum distal to the hypospadiac urethral meatus.

The severity of hypospadias is graded upon the position of the urinary meatus and the extent of ventral penile angulation. Severity rises as the distance of the displaced urethral opening increases from the normal position at the tip of the glans and with increasing penile curvature. Hypospadias is classified into three grades, depending on the location of the meatus: first degree/mild (glandular or coronal urethral opening), second degree/moderate (in the penile shaft) and third-degree/severe (urethral opening within the scrotum or perineum).

The exact etiology of the disorder remains unknown. It seems that the origin of hypospadias is multifactorial; several risk factors have been identified such as genetic predisposition, placental insufficiency, and substances that interfere with natural hormones, endocrine disruptors [3–6].

Up to 40% of hypospadiac fetuses have associated upper urinary tract anomalies. Cryptorchidism and inguinal hernias are the most common extragenital anomalies, which are found in 7–10% of all hypospadias cases [7].

In 5–10% of cases there are extragenital malformations such as heart defects, cleft palate, neural tube defects, and anorectal malformations. It has also been described in the context of various syndromes, such as XXY, XXXXY, trisomies 13 and 18, Fraser syndrome, the Smith-Lemli-Opitz, Opitz-Frias Wolf-Hirschhorn, and (4p−) [5].

Attention should be paid in detecting abnormalities in the external genitalia during second trimester ultrasound. Conventional sonographic criteria for the diagnosis of hypospadias are a small penis, with a blunted bulbous tip and two parallel echogenic lines at the top, representing the lateral folds of the foreskin. This was called by Meizner [8] the “tulip sign” for the diagnosis of severe hypospadias. This sign could be seen in our case (Figure 1).

However, in certain cases, especially when the testicles are undescended, it is difficult to distinguish between external female genitalia and severe hypospadias. So ambiguous genitalia would be included as main differential diagnosis, congenital adrenal hyperplasia being its primary cause. The sonographic findings of these virilized female fetuses are a clitoromegaly, which can mimic a penis, and enlarged adrenal glands bilaterally with discoid morphology [9]. This diagnosis can be ruled out by obtaining male karyotype, as in the case of our article.

The sonographic visualization of the fetal micturition is also important for its role in the diagnosis of hypospadias [10]. In a normal male fetus, the urinary stream is seen to escape from the tip of the penis. In hypospadias the urinary stream occurs in a direction perpendicular to the shaft of the penis from the ventral part, as in our case where we obtain the micturition after external stimulation (Figure 3).

Although 2D ultrasound is still the gold standard for diagnosing fetal genital abnormalities, prenatal diagnosis using 3D has been reported previously [11, 12]. A detailed volumetric ultrasound examination improves diagnosis and gives us information of anatomic structural relations, providing more detailed and realistic images, as shown in this case (Figure 2). By 3D representation, the external genitalia are shown with a great clarity of structures and their anatomical relation which confirm the diagnosis of third-degree hypospadias. In this case, despite the fact that severe hypospadias with chordee had been diagnosed, we detected an almost complete and surplus foreskin dorsally which is considered a favorable factor for obtaining good aesthetic and functional results with surgical reconstruction [13, 14]. Therefore, the multiplanar representation of the genital area allows us to obtain a better understanding of hypospadias and to get proper parental counseling from pediatric surgeons, with the possibility to report the prognosis and future treatment.

3D ultrasounds are a very interesting tool for diagnosis and clinical evaluation in prenatal diagnosis of hypospadias.
